# Examining the Osmotic Response of *Acidihalobacter aeolianus* after Exposure to Salt Stress

**DOI:** 10.3390/microorganisms10010022

**Published:** 2021-12-23

**Authors:** Melissa K. Corbett, Liam Anstiss, April Gifford, Ross M. Graham, Elizabeth L. J. Watkin

**Affiliations:** 1 Curtin Medical School, Curtin University, Perth, WA 6853, Australia; liam.anstiss@graduate.curtin.edu.au (L.A.); april.gifford@curtin.edu.au (A.G.); rmgraham@curtin.edu.au (R.M.G.); e.watkin@curtin.edu.au (E.L.J.W.); 2 Curtin Health Innovation Research Centre, Curtin University, Perth, WA 6853, Australia

**Keywords:** osmolytes, salt tolerance, ectoine, bioleaching

## Abstract

*Acidihalobacter aeolianus* is an acidophilic, halo-tolerant organism isolated from a marine environment near a hydrothermal vent, an ecosystem whereby levels of salinity and total dissolved salts are constantly fluctuating creating ongoing cellular stresses. In order to survive these continuing changes, the synthesis of compatible solutes—also known as organic osmolytes—is suspected to occur, aiding in minimising the overall impact of environmental instability. Previous studies on *A. aeolianus* identified genes necessary for the accumulation of proline, betaine and ectoine, which are known to act as compatible solutes in other halophilic species. In this study, the impact of increasing the osmotic stress as well as the toxic ion effect was investigated by subjecting *A. aeolianus* to concentrations of NaCl and MgSO_4_ up to 1.27 M. Exposure to high concentrations of Cl^−^ resulted in the increase of *ectC* expression in log-phase cells with a corresponding accumulation of ectoine at stationary phase. Osmotic stress via MgSO_4_ exposure did not trigger the same up-regulation of *ectC* or accumulation of ectoine, indicating the transcriptionally regulated response against osmotic stress was induced by chloride toxicity. These findings have highlighted how the adaptive properties of halo-tolerant organisms in acidic environments are likely to differ and are dependent on the initial stressor.

## 1. Introduction

The exploitation of microorganisms and their capabilities for biotechnological process is widespread in many industries and underlies the foundation of biomining operations. The microorganisms favoured in biomining operations oxidise iron and sulphur at low pH, resulting in the solubilisation of sulphide minerals. These acidophiles, whilst very good at mineral solubilisation, are typically sensitive to salinity [1,2], which can restrict biomining operations in areas where fresh water is limited. The low tolerance to salinity is attributed to the disruption of the chemiosmotic barrier by chloride ions moving into the cytoplasm. For acidophiles to maintain a near neutral cytoplasmic pH in highly acidic environments, establishment of a transmembrane potential at the cell wall is necessary whereby potassium cations are pumped into the cell creating a positive charge on the intracellular side of the membrane, with a negative charge on the outside [3,4]. Chloride ions can pass this barrier and accumulate, neutralizing the transmembrane potential thereby limiting the proton motive force and the accompanying cytoplasmic acidification resulting in cell death [5].

With the increasing scarcity of fresh water, growing demand within bioleaching operations for microorganisms capable of withstanding both acidic and saline conditions is intensifying. Additionally, pyritic ores are also know to contain high levels of chloride ions [6] which could also lead to the decreased recovery of minerals of interest. Unless these total dissolved solids (TDS) are removed from both the ore and water sources prior to leaching activities, they may accumulate to levels that are inhibitory to microbial action; therefore the overall profit margin from these operations is foreseen to decrease making the process uneconomical.

Halophiles are naturally salt-tolerant archaea and bacteria that have evolved to reside and flourish in saline rich environments [7], with different halophilic species having different salt tolerance levels. Recently, there have been significant advances in understanding the physiological mechanisms and gene expression changes that occur in halotolerant organisms [8,9] with a growing number of publications focussing on bioleaching organisms capable of tolerating high levels of salt [10,11,12]. Bioprospecting for iron-sulphur oxidising acidophilic halophiles in acidic waterways with high levels of TDS [13,14,15] lead to the discovery of what is now called the *Acidihalobacter* genus. The genus was established in 2015 [16] and to date has four species with documented differing NaCl tolerances. *A. aeolianus* (DSM 14174) was initially isolated from an acidic hydrothermal vent onshore of the Aeolian Island of Vulcano [17], along with two other species, *A. prosperus* (DSM 5130) and *A. ferrooxydans* (DSM 14175). *A. yilgarnensis* (DSM 105917) was isolated from an acidic salt drain in Western Australia [18]. Physiological experiments conducted following isolation has demonstrated that all species in this genus are sulphur-iron oxidising, acidophilic bacteria with pH requirements between 1.8 and 2.5 and salt tolerances between 0.05 M and 1.27 M of NaCl.

How these species and other halotolerant organisms thrive in low pH and high salt has been of great interest as salinity is responsible for evoking cellular distress through the process of osmotic stress and/or by the accumulation of toxic ions. Following exposure to low levels of osmotic stress, a biphasic response in bacteria is observed [19], initially involving the intake of inorganic ions, primarily K^+^ and a counter anion (glutamate), stabilising the cell [20]. Ongoing or continued high levels of osmotic stress will result in the accumulation of a potassium-glutamate complex, triggering the initiation of the organic osmolyte response [4,21]. Bacteria can synthesise organic osmolytes de novo or scavenge them from the environment [22] with solubilisation occurring in the cytoplasm. These solutes comprise amino acids (proline and taurine), amino acid derivatives (ectoine and hydroxyectoine), betaines (glycine betaine), sugars, and sugar derivatives including trehalose. As these solutes carry a net zero charge, accumulation in the cytoplasm is possible without impact on biomolecules or intracellular machinery, enabling the cell to equalise intracellular solute concentrations with the extracellular environment [23], resulting in the reduction of osmotic stress [11,19]. The ability to accumulate and utilise the osmolyte response differs between species, and for acidophiles this can be determined by an organisms’ tolerance to chloride stress [20].

Numerous organisms employed in bioleaching operations have demonstrated at least some capacity to employ the osmolyte response to tolerate salt stresses, including *Acidiphilum cryptum* (hydroxyectoine) [24], *Acidithiobacillus ferrooxidans* (proline and betaine) [25] and *Acidihalobacter prosperus* (ectoine) [11]. Proteomic investigations into *Acidihalobacter* genus has revealed a reliance on the production of various osmolytes to protect and support continued cell metabolism and turgor [26]. In response to increasing NaCl concentrations, synthesis of ectoine, betaine and proline were notably upregulated, with supporting proteomic data highlighting an increase of the ABC transporter specific to ectoine in *A. prosperus*, and a 423 fold increase of l -ectoine synthase (from *ectC*) in *A. aeolianus*, an enzyme necessary for fabrication of ectoine [11,26]

From the *Acidihalobacter* genus, *A. aeolianus* has the highest chloride tolerance reported at 1.27 M and has previously demonstrated a measurable difference in the accumulation of ectoine when exposed to low (0.14 M) and high (0.85 M) NaCl [26]. The same study demonstrated that accumulation of ectoine is the largest response mechanism employed to tolerate increasing salt concentrations; however, it is unknown if this is in response to the total toxic ion increase or only in response to changes in osmotic pressure. To address this issue and determine whether the stress response employed was due to the toxic ion effect or from osmotic stress, we conducted experiments on *A. aeolianus* with MgSO_4_ and NaCl. As sulphate anions are non-membrane permeable, growth in the presence of MgSO_4_ will result in osmotic stress without the accompanying toxic ion effect of membrane permeable chloride, and acts as a vehicle for testing osmotic stress alone. By understanding whether the impact of salt stress is from toxic ions or by osmotic stress, we can better approach the processes of adapting halo-tolerant acidophilic iron-oxidizing microorganisms to industrial bioleaching.

## 2. Materials and Methods

### 2.1. Culture Maintenance

*Acidihalobacter aeolianus* DSM 14174 was obtained from Deutsche Sammlung von Mikroorganismen und Zellkulturen GmbH (DSMZ) and grown in basal salts medium (BSM) (L^−1^): (NH_4_)_2_SO_4_ 0.4 g, MgSO_4_·7H_2_O 0.5 g, KH_2_PO_4_ 0.2 g and adjusted to pH 2.0 with H_2_SO_4_. The media was supplemented with 2 mL trace salts solution (L^−1^) MnCl_2_·2H_2_O, 62 mg, ZnSO_4_·7H_2_O, 68 mg, CoCl_2_^·^6H_2_O, 64 mg, H_3_BO_3_, 30 mg, Na_2_MoO_4_, 10 mg, CuCl_2_·2H_2_O, 66 mg. Filter sterilised (0.2 µm) FeSO_4·_7H_2_O (13.9 gL^−1^) and K_2_S_2_O_3_ (0.15 gL^−1^) were added after autoclaving. Culture flasks were seeded with an initial cell density of approximately 2.2 × 10^7^ cfu/mL in 200 mL of BSM. Cultures were maintained at 30 °C with shaking at 100 rpm.

### 2.2. Induction of Salt Stress Conditions

The effect of increasing salt stress on the growth of *A. aeolianus* was tested with concentrations of 0.05, 0.1, 0.25, 0.45, 0.85 and 1.27 M NaCl or MgSO_4_ in triplicate. *A. aeolianus* has a minimum salt requirement of 0.05 M NaCl, which was added to the media used to investigate MgSO_4_ stress. Effects of salt stress were measured by pH, redox potential and ferrous iron oxidation with samples taken 0, 24, 48, 56, 72 and 96 h post inoculation. Samples were filter sterilised (0.2 μm) and iron oxidation, redox and pH determined through methods described in Govender et al. [27] and Khaleque et al. [28].

### 2.3. RNA Isolation and cDNA Synthesis

*A. aeolianus* was harvested after 24 h for 0.25 M NaCl (low) and MgSO_4_ cultures and after 48 h for those at 0.85 M (high). Cells were pelleted by centrifugation at 20,000× *g* for 40 min at 4 °C, resuspended in 1 mL of 1:1 phosphate buffer saline (PBS) pH 2.0 and Buffer A [29], then centrifuged for 20 min at 20,000× *g* 4 °C. The supernatant was discarded, and the wash repeated. Cells were resuspended in 500 μL Buffer A and 30 μL of 100 mg mL^−1^ lysozyme (Sigma), mixed, then incubated at 37 °C for 20 min. Samples were sonicated using a water bath (Diagenode Bioraptor^TM^ Plus) for 20 cycles of 10 s on high and 20 s off. To the preparation, 100 μL of 20% sodium dodecyl sulfate (SDS; Astral), 15 μL β-mercaptoethanol (Bioline, Memphis, TN, USA) and 60 μL of 50 mg mL proteinase K (Promega) were added. Samples were inverted and incubated at 55 °C for 45 min. A further addition of 100 µL of 20% SDS and 500 µL of acidified phenol-chloroform-isoamyl alcohol 25:24:1 (pH 4.5) was performed, and then centrifuged at 16,000× *g* for 15 min at 4 °C.

The aqueous layer was retained, and the extraction repeated with an equal volume of phenol-chloroform-isoamyl alcohol 25:24:1 (pH 4.5) and centrifuging at 14,000× *g* for 10 min at 4 °C. The aqueous portion was again placed into a fresh tube with the addition of 1 volume of 100% isopropanol alcohol followed by 0.1 volume of 3 M Na-Acetate (pH 5.2) and incubated overnight at −20 °C to precipitate the RNA. The RNA was pelleted by centrifugation at 16,000× *g* 4 °C for 30 min. The pellet was rinsed with 500 μL cold 70% ethanol and centrifuged at 14,000× *g* 4 °C for 10 min. The supernatant was decanted and the RNA pellet air dried, resuspended in 30 µL DNase 1 solution (New England BioLabs) and treated according to the manufacturer’s protocol to remove any trace of genomic DNA. RNA purity was determined by optical density (OD) 260/280 and 260/230 ratios. All samples passed quality control with the ratio OD260/280 between 1.9 and 2.2 and ratio OD260/230 < 2.0. The quantity of RNA was assessed using a Qubit™ 2.0 Fluorometer (Life Technologies, Thermo Fisher Scientific Inc., Waltham, MA, USA) with the Qubit™ RNA HS Assay Kit (Life Technologies, Thermo Fisher Scientific Inc., Waltham, MA, USA). A 500 ng sample of RNA from each culture condition was reverse-transcribed into cDNA using a Sensifast cDNA synthesis kit (Bioline, Eveleigh, Australia) following the manufacturer’s protocol. cDNA was stored at −80 °C until further use.

### 2.4. Candidate Reference Genes Primer Construction

RT-qPCR primers for the three candidate reference genes, DNA gyrase subunit A (*gyrA),* tryptophanyl-tRNA synthetase (*WARS*) and alanyl-tRNA synthetase (*AARS*) and one target gene l-ectoine synthase (*ectC*) were designed using Geneious (version 10.2.6). All had a length of 20–25 bases and a theoretical T_m_ of 55 °C. The primers were checked for gene-specific binding using the genome *A. aeolianus* (CP017448.1) deposited at the DDBJ/EMBL/GenBank. The primer sequences are listed in Table 1.

### 2.5. Quantitative Real Time PCR

All RT-qPCR’s were performed in a CFX Connect™ real-time thermocycler (Biorad) with the SensiFAST SYBR^®^ Lo-ROX kit (Bioline). Each reaction was performed in triplicate with a total reaction mixture of 20 μL final volume containing 1 μL diluted cDNA, 0.3 μM of each primer, 10 μL of SensiFAST SYBR^®^ Lo-ROX master mix, and RNase-free water. The qPCR cycling conditions were an initial denaturation step at 95 °C for 2 min, followed by 45 cycles of denaturation at 95 °C for 15 s, annealing at 55.5 °C for 15 s, and extension at 72 °C for 15 s followed by a final extension at 72 °C for 30 s. Product specificity was confirmed by melt-curve analysis. The threshold cycle (Ct) was automatically calculated by the CFX Manager software (Biorad). Relative gene expression using only a single reference gene was determined by the ∆∆Ct method of Pfaff [30], and for multiple reference genes by the methods of Vandesompele et al. [31] and Hellemans et al. [32]. The mean of the low MgSO_4_ was used as the control group for the calibration of relative gene expression. Statistical significance between groups was determined with a Mann–Whitney test using GraphPad Prism version 8.4.3 (GraphPad Software, La Jolla, CA, USA).

### 2.6. Statistical Analysis of Gene Expression

The evaluation of reference gene validity under salt stress conditions was performed using the algorithms BestKeeper [30] NormFinder [33], GeNorm [31] and RefFinder [34]. BestKeeper analysis was performed using the raw Ct values, whereas calculations performed by both NormFinder and GeNorm depended on relative quantities (RQ). The RQ values for the reference genes were calculated through 2^−∆Ct^ method: ∆Ct = Ct of sample—minimum Ct. Overall reliability and stability statuses were created with RefFinder.

### 2.7. Metabolite Extraction

Cell lysate was extracted as described in Khaleque et al. [26] with some modifications. Cultures were harvested at stationary phase (72 h for low salt and 96 h for high salt cultures) and collected by centrifugation at 20,000× *g* for 40 min at 4 °C. Cell pellets were suspended in 1 mL of media containing corresponding molar salt concentrations. The samples were centrifuged at 14,800× *g* for 30 min, the supernatant discarded and the pellet resuspended in 0.1 mL TE buffer (pH 7.4) with 1% SDS and 1 mg mL^−1^ lysozyme, incubated at room temperature for 20 min then incubated at 95 °C for 10 min. Samples were cooled before adding 0.4 mL of lysis buffer (7 M urea, 2 M thiourea, 1% SDS, 40 mM Tris pH 7.4 and 1 mM EDTA), briefly mixed, then sonicated in a sonicating water bath for 100 rounds of 20 s followed by 20 s rest. A volume of 0.5 mL chloroform was added to each sample, mixed and centrifuged at 14,800× *g* for 10 min. The aqueous top layer of the sample was collected (~0.5 mL) in auto sampling vials for analysis.

### 2.8. Analysis of Osmolyte Content

The total osmolyte content was quantified by liquid chromatography mass spectrometry (LC-MS) on extracted samples using an Agilent 6540 UHD Accurate Mass QToF LCMS with 1290 HPLC System. Sample injection volumes were 1 µL, auto-loaded and run on an Agilent InfinityLab Poroshell 120 HILIC-Z column, (2.1 × 100 mm) (1.9 µm × 120 Å pore size) with a pre-programmed ID tag. Separation solvents were solvent A (20 mM NH_4_HCO_2_ in 100% ddH_2_O, pH adjusted to 3.0 with HCOOH) and solvent B (20 mM NH_4_HCO_2_ in ddH_2_O and 1:10 acetonitrile solution, pH adjusted to 3.0 with HCOOH). Electrospray ionisation mass spectrometry (ESI-MS) source settings are listed in Table 2. Aqueous external standards of betaine, proline, ectoine and hydroxyectoine (Sigma-Aldrich), dissolved in ddH_2_O and serial diluted to 0.36 µg mL^−1^ were used to calibrate the data. Acquired data were processed by MassHunter Qualitative Analysis B.05.00. Results were calculated using ratios of the peak area of the analytes to their standards. Results from the analysis were standardised and recorded as a response factor via the following equation.

Response factor (RF) = ((PA_s_/PA_x_)/T_c_) × 10^9^ whereby PA_s_ is the peak area of sample, PA_x_ is peak area of known standard signal and T_c_ is the total cell count of the sample. Significance of differences between the samples were determined using two-sample *t*-tests.

## 3. Results

### 3.1. Exposure of Acidihalobacter aeolianus DSM 14174 to Increasing Concentrations of NaCl and MgSO4 Impacts Growth and Iron Oxidation Rates

**Figure 1:**
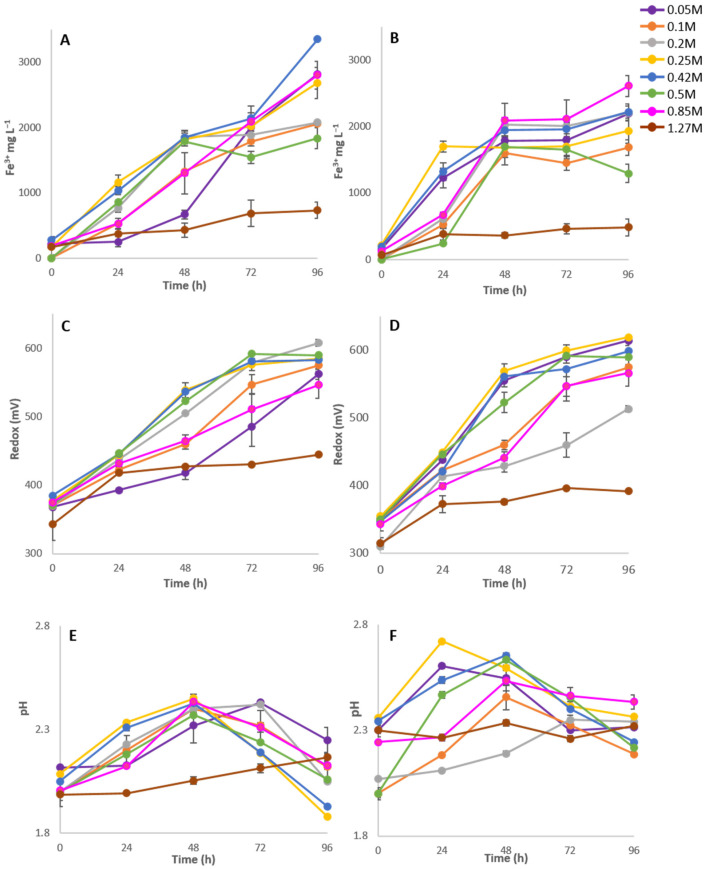
Ferric iron concentration, Redox potential and pH under increasing concentrations of NaCl (**A**,**C**,**E**) or MgSO_4_ (**B**,**D**,**F**) in *Acidihalobacter aeolianus*. Values are the average of biological triplicates and include the standard error.

To assess the adaptive response of *A. aeolianus* DSM 14174 to osmotic stress, growth under various salt concentrations of NaCl and MgSO_4_ was first examined by monitoring ferric iron generation, redox potential and pH. In cultures containing 0.05, 0.85 and 1.27 M NaCl, initial generation of Fe^3+^ slightly lagged behind that of 0.25 and 0.42 M at 24 h (Figure 1A). For example, Fe^3+^ was 2.2-fold higher in 0.25 M NaCl compared to 0.85 M NaCl at 24 h (*p* < 0.01). Although not statistically different, this difference was also recorded with a lower Eh value (Figure 1C) and a small change in pH (Figure 1E) for the same cultures at 24 h. This confirms data previously reported on this strain [28] and indicates that even though cultures of *Acidihalobacter* have a minimum requirement of 0.05 M salt for survival, a greater concentration could improve growth rates. Those cultures with 0.1 to 0.5 M NaCl showed very little difference in rates of ferric iron generation and redox potential (Figure 1) and as such, RNA and protein experiments work was performed on the 0.25 M cultures and will be referred to as ‘low stress’. After 48 h, the 0.85 M NaCl culture demonstrated an increase in Fe^3+^ production that was not seen in the 0.05 M culture until 72 h. Minimal generation of Fe^3+^ was recorded for cultures in 1.27 M NaCl, with little change in Eh or pH logged. The ‘high stress’ point for these experiments was therefore deemed to be at 0.85 M as it was the highest salt concentration that still showed high levels of oxidation occurring in both NaCl and MgSO_4_ (Figure 1).

Cultures in 0.25 M MgSO_4_ mimicked those of NaCl at 24 h and demonstrated a greater level of Fe^3+^ than any other concentration being examined at the same time (*p* < 0.001 between 0.25 M and 0.85 M MgSO_4_). After 48 h, cultures grown in the presence of 0.05 through to 0.85 M MgSO_4_ showed similar levels of iron oxidation (Figure 1B) however, the Eh values demonstrated greater variability at the same time point (Figure 1D). Cultures subjected to 1.27 M MgSO_4_ show little iron oxidation, no change in pH or alteration of the redox potential.

To identify the exponential growth phase of *A. aeolianus* for expression studies of *ectC*, ferric iron, redox and pH results were used as a measure of growth as manual cell counting is often unreliable [28]. Ferric iron concentration is a representation of energy used; however, it does not necessarily correlate with an increase in cell numbers as energy could be funnelled towards other cellular processes in response to stress [35,36]. As such, exponential growth was estimated at 24 h for cultures under low stress NaCl and MgSO_4_ at 48 h for those under high stress. Accordingly, cultures were harvested for RNA at these time points.

### 3.2. Reference Validation and Expression Stability of Reference Genes under Low and High Salt Conditions

For this study, four genes (*gyrA*, *WARS*, *AARS* and *rrs*) were selected and evaluated as potential candidate reference genes to act as controls, enabling the relative expression of *ectC* to be calculated under the different salt conditions. The *rrs* gene was removed from this study following examination of literature that documents it as an unreliable reference gene due to high copy number in some species. Dissociation curves revealed only single peaks and single amplicons were confirmed by gel electrophoresis (data not shown). Amplification efficiency was determined by the slope of calibration and for all candidate genes, the PCR efficiencies were within the acceptable range [37], except for *gyrA*. The PCR efficiency for *gyrA* dropped to 88.2% (slightly below the acceptable low range marker of 90%) due to increasing the annealing temperature from its optimum at 54 °C to 55.5 °C (as was calculated during primer design and seen in Table 1). Conversely, the relative gene expression calculation methods employed accounted for the primer efficiencies [30,31,32] and all correlation coefficients (R^2^) were satisfactory (>0.99) verifying the quality of the primers. In order to select the best candidate control genes for use in relative expression, it was necessary to ensure that the reference gene did not change in response to altered environmental conditions. Expression stability of each of the reference genes under different salt conditions was evaluated with Best Keeper, NormFinder, GeNorm and RefFinder with outcomes depicted in Table 3. Detailed analysis for the algorithms are detailed in the supplementary data (Tables S1 and S2). Due to the consistent high ranking of the *WARS* gene regardless of the algorithm chosen, *WARS* was selected as the most stable reference gene for normalising *ectC* expression under all salt conditions examined.

### 3.3. mRNA Level of ectC Increase under Low and High NaCl Conditions

**Figure 2:**
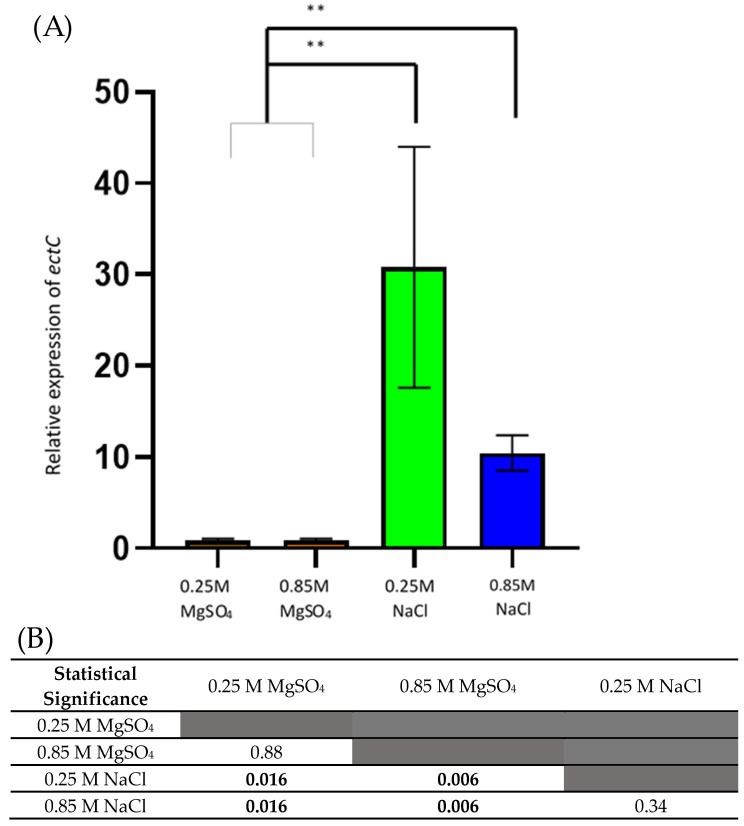
(**A**) Relative expression levels of *ectC* in *Acidihalobacter aeolianus* normalised to the reference gene *WARS*. Data are shown as mean ± standard error of the mean (SEM). ** Indicates statistically significant difference as given in (**B**).

Expression of *ectC* was assessed at 24 h post exposure to low salt NaCl and MgSO_4_ (0.25 M) as cultures were estimated to be in exponential growth at this time point (Figure 1). High salt NaCl and MgSO_4_ (0.85 M) cultures were assessed at 48 h for the same reason. The greatest amount of *ectC* expression was detected in cultures of low NaCl, a 34-fold increase in transcription triggered by NaCl exposure after 24 h (Figure 2A). A similar result was also detected in the higher salt concentrations whereby *ectC* expression in the high NaCl cultures was 11 times greater than the same concentration of MgSO_4_ at the same time. A statistically significant difference in the expression of *ectC* was recorded between the two salt types, but not between the concentrations of each salt (Figure 2B).

### 3.4. High Salt Cultures Accumulate the Compatible Solutes Betaine and Ectoine

**Figure 3:**
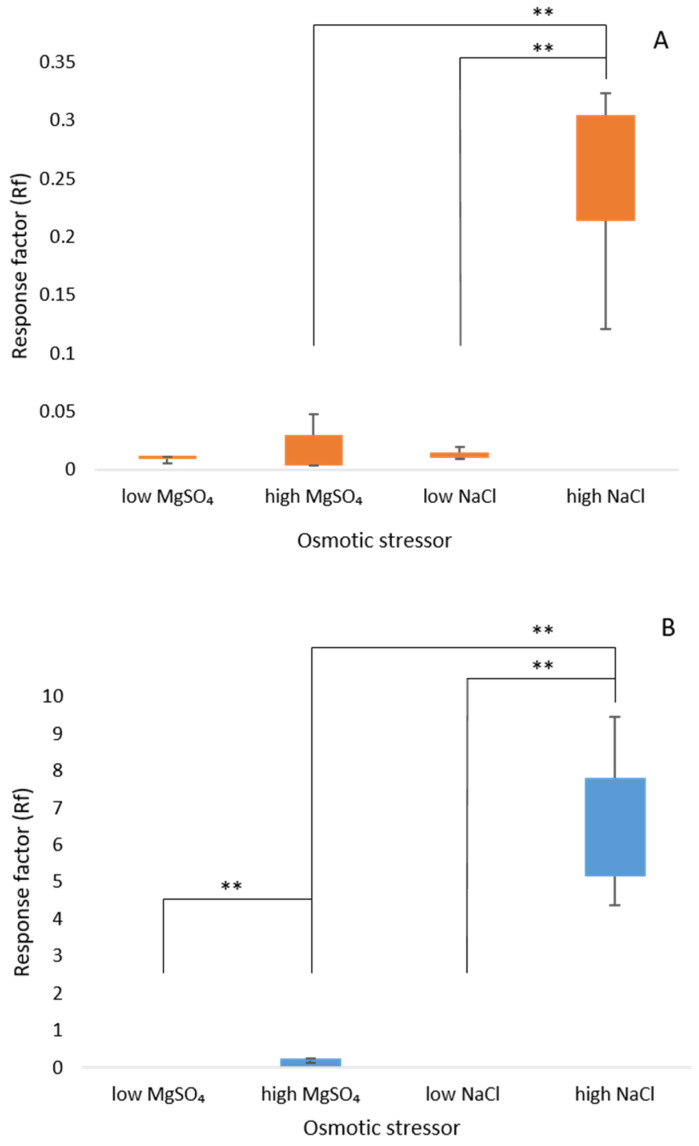

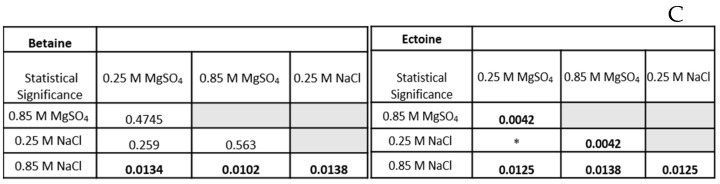
Standardized response of (**A**) betaine and (**B**) ectoine in *Acidihalobacter aeolianus* grown in the presence of either NaCl or MgSO_4_ from three biological replicates. * Values were 0 for this comparison. ** Indicates statistically significant difference as given in (**C**).

Betaine was detected in all cell lysate samples—low (0.25 M) and high salts (0.85 M). The greatest amount was recorded in the high NaCl samples compared to low NaCl or high MgSO_4_ (both *p* < 0.01; Figure 3A). Ectoine was only detected in cell lysates from cultures grown in high NaCl and MgSO_4_ with concentrations in low salt below threshold. Other known osmolytes, proline, glycine, taurine and hydroxyectoine were not detected in any samples. There was no statistically significant change in the response factor for betaine expression in low and high MgSO_4_, (Figure 3A,C) whereas high NaCl showed a 17.4-fold increase in betaine compared to the low salt culture (*p* < 0.01). Ectoine accumulation was significantly greater in high NaCl than in MgSO_4_ (30.9 fold) or low NaCl (both *p* < 0.01; Figure 3B).

## 4. Discussion

The community of known acidophilic halotolerant organisms is a small one and evolution of the different mechanisms designed to cope with the environmental stresses are of interest. To date, only a few organisms used in bioleaching operations can tolerate high levels of chloride stress, with *Acidihalobacter* isolates capable of iron oxidation even when concentrations of chloride are higher than seawater (0.53 M). When administered at equal concentrations, NaCl and MgSO_4_ exert the same level of osmotic pressure onto an organism in a solution. However, sulfate (SO₄^2−^) is not membrane permeable and as such it cannot cross into the cytoplasm to neutralise the positive membrane, therefore it cannot cause a toxic ion effect [35,36]. This allows for MgSO_4_ to be used to assess osmotic stress versus NaCl as the toxic ion effect contributor.

*A. aeolianus* demonstrated the ability to oxidise iron at a faster rate under MgSO_4_ than under NaCl as a rapid rise in ferric iron concentrations was recorded, and by 48 h, Fe^3+^ concentrations had plateaued even at high salt concentrations. Those cultures subjected to NaCl stress had not reached maximum Fe^3+^ generation even by 96 h; therefore increased osmotic stress signalling had a negative impact on iron oxidation. Additionally, as the concentration increases from low to high under both salt conditions, a distinct latency in iron oxidation is observed and although NaCl cultures exhibited higher ferric iron concentrations by 96 h compared to MgSO_4,_ it could be attributed to a sensitivity error of the assay. All cultures contained 13.9 g L^−1^ of FeSO_4_, and as ferric iron is preferred over sulphate as an electron donor, the plateau of ferric ion oxidation in MgSO_4_ is indicative of complete ferrous iron consumption and a switch to sulphur metabolism [38,39].

A rapid drop in pH (generation of sulfuric acid) following the plateau in ferric iron concentration for MgSO_4_ stress cultures supports the theory of sulphur oxidation (Figure 1). Under both salt conditions, the optimal bioleaching rate was at 0.25 M depicted by the fastest rise in ferric iron concentration, redox potential and earliest indication of sulphur oxidation (Figure 1). This confirms the previous studies that observed 0.25 M be the optimal NaCl concentration and is the first study to determine the optimal concentration for MgSO_4_ [40,41].

The oxidation of iron by *A. aeolianus* is impaired when salt concentrations are 1.27 M and above. Previous studies that characterized this species found a similar result [40], however the inability to oxidise iron above 1.27 M MgSO_4_ was unexpected as many acidophiles are capable of tolerating higher MgSO_4_ concentrations than NaCl. *Acidithiobacillus ferrooxidans,* a model bioleaching organism, is inhibited by 0.1 M NaCl but can tolerate 0.35 M MgSO_4_, whereas *Acidiphilium cryptum* DSM2389 and *Acidiphilium acidophilum* DSM700 ^T^ are inhibited by <0.17 M of NaCl, but tolerate up to 0.47 M MgSO_4_ [42]. Whilst this MgSO_4_ concentration tolerance of *A. aeolianus* is lower than expected given the trend seen in other acidophiles, 0.85 M is still the highest MgSO_4_ tolerance concentration recorded for a bioleaching acidophile. In order to determine the exact threshold for *A. aeolianus* iron oxidation inhibition (>0.85 but <1.27 M MgSO_4_) further research is required, and if *A. aeolianus* tolerance of MgSO_4_ is less than that of NaCl, this would be a unique characteristic that demarks it from other acidophiles. From the data acquired, it can be found that the bioleaching capabilities of *A. aeolianus* are less inhibited by MgSO_4_ than NaCl.

### 4.1. ectC Expression Is Dependent on the Salt Stress Type

When studying the impact of osmotic stress on *A. aeolianus*, our results showed that exposure to increasing concentrations of MgSO_4_ hindered iron oxidation rates, however, no significant increase in *ectC* transcription correlated with this finding, as the relative expression of *ectC* in low MgSO_4_ was 0.89 and at high MgSO_4_, only 0.91. In comparison, both low and high levels of NaCl saw an increase in *ectC* expression, with the low concentration three times higher than that of the high culture. As many acidophilic bacteria are known to be very osmotolerant, growing in media containing high concentrations of sulphate salts (>1 M) [43], it appears that the toxicity of chloride anions (as opposed to the comparative nontoxicity of sodium cations) is responsible for triggering the production of ectoine. Expression of *ectC* in response to the chloride anions was 34 times greater than that of MgSO_4_ cultures at low concentrations and 11 times greater at high concentrations. This phenomenon indicates that *A. aeolianus* utilizes a different cellular response to osmotic stress depending on the form in which the osmotic stress occurs and without a toxic ion event occurring, ectoine is not used to aid in balancing the osmotic pressure.

### 4.2. Osmolyte Accumulation in A. aeolianus Is Caused by NaCl Stress

Culturing of *A. aeolianus* requires a low level of total salts for growth. To the basal salts media employed, no organic solutes or osmolyte precursors are added, so detection of osmolytes in cell lysates points to de novo synthesis of these compounds in response to environmental stresses. Based on previous research conducted by this group [26] production of ectoine was expected under salt stress conditions, but an interesting discovery was the additional production of betaine in all samples examined. Halophilic and halotolerant bacteria are known to utilise betaine as an osmolyte [44] due to its high solubility and low protein interaction at high concentrations. Interestingly this study found that betaine, even though present in all samples, did not accumulate under high MgSO_4_ conditions with concentrations resembling that of the low salt cultures, whereas a 17.4 fold difference was seen between low and high NaCl cultures. Potentially, NaCl is responsible for instigating a multi-factorial stress response, resultant in numerous stress adaptation pathways being enacted. Whereas, MgSO_4_, with SO_4_^−2^ unable to cross the cell membrane, it is likely that a minimalist or more energy conserved pathway maybe engaged.

Betaine accumulation post exposure to NaCl has been observed in other halotolerant organisms [45,46] with detection of betaine synthesis enzymes choline dehydrogenase and betaine-aldehyde dehydrogenase. For *A. aeolianus,* betaine concentrations were low in comparison to the other osmolyte ectoine, which highlights the fact that betaine may play a minimal role in stress reduction or accumulation of betaine maybe an initial-stress response only. Synthesis of betaine via the *bet* gene family [45,47,48] in other organisms is dependent on the precursor choline [49], usually exogenously sourced. In these experiments, no chloine was added to growth media. A small number of extreme halophiles have demonstrated de novo betaine synthesis [50], reliant on the methyltransferase enzymes to methylate glycine, however analysis of the *A. aeolianus* genome for genes associated with this methyltransferase pathways failed to yield any homologous sequences. Further analysis of the *A. aeolianus* genome however does reveal the presence of proline/glycine-betaine periplasmic transporters and permeases *proX, proV, proW* and *opuAC* necessary for the process of osmolyte accumulation. As betaine was detected in *A. aeolianus* cell extracts without exogenous choline present, albeit at low levels, we theorise that it is not the primary osmolyte employed to tolerate osmotic stress and may in fact have an entirely separate role, however the production of betaine by *A. aeolianus* does warrant further study.

Under high stress conditions elicited by NaCl, ectoine accumulation was noticeably greater than that of betaine (25.5 fold increase) and not detected in either low NaCl or MgSO_4_ conditions. The accumulation of ectoine by other extreme halophiles occurs by the conversion of l -aspartate to ectoine [51] under the control of gene cluster *ectABC,* which is present in *A. aeolianus* as well as the ectoine transporter and permeases cluster *ehuABCD* [12]. Ectoine production is growth-phase dependent in the halophilic model organism *Halobacillus halophilus* with maximal accumulation occurring during the stationary phase of growth [52]. For this reason, cell lysates experiments were conducted later than those on *ectC* expression at exponential growth. The amassing of low levels of betaine across all salt stress concentrations combined with the accumulation of ectoine only under high NaCl stress, points to an osmolyte switching strategy whereby the composition of the compatible solutes pool is contingent on the level of salt stress experienced. This is beneficial to *A. aeolianus* as ectoine is one of the more energy expensive osmolytes [53], so production of this compound only under extreme stress conditions is likely to allow for energy conservation. As cytoplasmic acidification also disrupts ATP generation [35,54,55] protection of proteins and enzymes becomes an essential step that is necessary for the continued production of ectoine.

For *A. aeolianus,* ectoine accumulation is up-regulated by salinity and could possibly be regulated by levels of betaine. Studies of this interaction have been conducted on *Chromohalobacter salexigens* [56] whereby the presence of exogenous choline results in the use of betaine over ectoine, even under high salt conditions, whereas when choline is absent, ectoine is employed as the major osmolyte. These results on *A. aeolianus* demonstrate a similar adaptation process, however it should be noted that the long-term reaction to osmotic stress by bacteria is complex and it is possible that more than one compatible solute can be synthesised and accumulated at a time [57].

### 4.3. Impact of Toxic Ion Effect vs. Osmotic Stress

As ectoine was not the only organic osmolyte identified in *A. aeolianus* when placed under stress (Figure 3, presence of betaine detected in both NaCl and MgSO_4_ cultures) it is conceivable that other osmolytes are being used to counteract the osmotic pressure resultant from high levels of sulfate cations, possibly below our levels of detection. With the combination of *ectC* gene expression and solute accumulation data, it becomes apparent that toxic ion stress has a greater role in triggering ectoine production than osmotic stress alone, however it is also likely that other mechanisms are contributing to the high levels of halotolerance seen in *A. aeolianus.* Outer membrane proteins and porins have been shown to decrease in concentration in *Acidihalobacter* [11,26] following exposure to high concentrations of NaCl, with a reduction in these proteins likely restricting chloride entry and thus decreasing the toxic ion effect. The unique halotolerance exhibited by *A. aeolianus* is likely to be reliant on numerous mechanisms, of which ectoine may play only a small part.

This study hypothesised that by increasing the salt concentration, more *ectC* and ectoine would be produced by *A. aeolianus,* however, protein and gene expression did not correlate between the salt concentrations tested. We speculate that discrepancy between transcript levels and protein concentrations was because high level of *ectC* transcripts after 24 h in low NaCl arose due to cellular responses from the initial salt shock, which in turn was lost from the high NaCl cultures after 48 h post exposure due to the constant level of salinity experienced. Further investigations are needed at greater time points to elicit the cellular reactions at both the gene and protein level to confirm the results observed in this study. Investigations into expression of glycine-betaine genes across the NaCl concentrations would also aid towards supporting the theory of multi-osmolyte response to toxic ion stresses.

## 5. Conclusions

This study highlights that ectoine is employed by *A. aeolianus* to mitigate the impact of high levels of chloride and is not just as a response to osmotic pressure in an environment rich in TDS. Previous research proposed that ectoine was a significant contributor to the unique tolerance of *Acidihalobacter* in high salt environments [12], and this work strengthens that finding noting that the osmolyte response maybe biphasic, with alternative adaptations utilised for low-moderate stresses.

To summarise, osmolyte accumulation occurs in *Acidihalobacter aeolianus DSM 14174* as hypothesised under high toxic ion stress and this method of accumulation is more comparable to that seen in halophilic bacteria rather than acidophiles. Our research is the first observation known to date, of which betaine and ectoine accumulation is utilised as an adaption to osmotic stress in an acidophile.

## Figures and Tables

**Table 1:** Selected candidate reference genes, corresponding product name, primer sequences, amplicon size (bp), PCR amplification efficiencies and the mean C_T_ values (±standard deviation) assessed in *A. aelioanus* cells under high and low salt. R^2^: correlation coefficient of the standard curve; E: PCR efficiency (%).

Gene	Gene Product	Primer (5′–3′)	Reference	AmpliconSize (bp)	Optimal Annealing Temperature (°C)	Standard Curve R^2^	E (%)
*ectC*	l-ectoine synthase	(F)ATGCAGATTGTGTTCTTCGTGC (R)GTGTGACGCACATTTGGTACAA	This study	152	55.5 °C	0.99	105
*WARS*	Tryptophanyl-tRNA synthetase	(F)GAGTTCCGTTCTGGTACCCAAT (R)GATGGCTCAGATCCTCGTAGTG	This Study	182	55.5 °C	0.99	100.0
*AARS*	Alanyl-tRNA synthetase	(F)GGTGCAGTTCAAGGATGTGTTC (R)CTTGAAGTAATCGCCGAAGCTG	This study	181	55.5 °C	0.99	114.2
*gyrA*	DNA gyrase subunit A	(F)GAAATGCGCCAGTCCTACCT (R)TRTACGCCTTGTTCCAKTCG	[17]	147	54 °C	0.99	88.2

**Table 2:** ESI-MS settings for osmolyte analysis on an Agilent 6540 UHD Accurate Mass QToF with an InfinityLab Poroshell 120 HILIC-Z column.

ESI-MS Source Settings			
Gas temperature	300 °C	Nozzle voltage	0 V
Drying gas	7 L·min−1	Fragmenter	125 V
Nebulizer	35 psig	Skimmer	65 V
Sheath gas	400 °C	OCT IRF V6p	750 V
Sheath gas flow	12 L·min−1	Column tempt	25 °C
Voltage cap	3000 V	Ion polarity	Positive

**Table 3:** Reference genes ranked by their expression stability calculated by BestKeeper, GeNorm, NormFinder and RefFinder.

Group	Gene	BestKeeper Rank	SD [±x-Fold]	GeNorm Rank	M-Value	Normfinder Rank	Stability Value	RefFinder Stability Rank	GeoMean
NaCl	*WARS*	3	2.57	3	1.306	1	0.898	2	1.73
	*gyrA*	4	2.87	4	1.547	4	1.282	4	4
	*AARS*	5	3.66	5	1.634	5	1.39	5	5
MgSO_4_	*WARS*	1	1.37	1	1.109	3	1.217	2	1.73
	*gyrA*	2	2.03	1	1.109	2	1.018	1	1.68

## Data Availability

The data presented in this study are available on request from the corresponding author.

## Supplementary Materials

The following are available online at https://www.mdpi.com/article/10.3390/microorganisms10010022/s1, Table S1. BestKeeper algorithm analysis. Table S2. BestKeeper algorithm analysis of *WARS* and *gyrA* under both NaCl and MgSO_4_ conditions.
