# Supplementary material for: Examining the Osmotic Response of Acidihalobacter aeolianus after Exposure to Salt Stress
Source: Microorganisms. 2021 Dec 23;10(1):22. doi: 10.3390/microorganisms10010022 (PMC8781986; doi:10.3390/microorganisms10010022)
Supplement: Supplementary file 1 [file microorganisms-10-00022-s001.zip › microorganisms-1488858-supplementary.pdf]

Supplementary Materials:

Validation of the reference genes for normalization of *ectC* expression

Expression studies with some of these genes has highlighted that their suitability as reference genes is questionable [1], especially the *16S* rRNA, whose high abundance of transcripts can often dwarf that of the rarer genes under investigation [2]. This effect has been seen in the acidophile *Acidothiobacillus ferrooxidans*, whose abundant expression of the 16S rRNA resulted in the under reporting of low copy transcripts [3]. For this reason we excluded it from our study. Three genes selected for this study whose presence was confirmed in the *A. aeolianus* genome (*WARS, AARS and gyrA*) were assessed for expression stability by BestKeeper, NormFinder, geNorm and RefFinder. The final rankings of the candidate reference genes were not identical when the different algorithms were employed, however the least stable gene (*AARS*) was consistently identified by all programs.

Both *gyrA* and *AARS* demonstrated a lack of stability, more so when subjected to NaCl than $MgSO_4$ highlighting the issue that it is difficult to identify ideal reference genes that have stable expression regardless of the physiological state. For this study Cl^- ions that can cross the cell membrane demonstrated disruption to the expression of core genes which further supports the idea that candidate genes must be validated in every physiological condition prior to use for RT-qPCR normalisation [4].

Table S1: BestKeeper algorithm analysis [Ct] = cycle quantification; Geo Mean [Ct] = geometric mean of Ct; Min and Max [Ct] = the extreme values of Ct; SD [Ct] = standard deviation of Ct; CV [% Ct] = coefficient of variance of Ct and expressed as percentage; Min [x-fold] and Max [x-fold] = the extreme values of expression levels presented as an absolute x-fold over or under-regulation coefficient; SD [± x-fold] = standard deviation of the absolute regulation coefficients, Coeff. of Corr.[r] = coefficient of correlation between each candidate and the BestKeeper index.

	NaCl			$MgSO_4$	
**Gene name**	***WARS***	***gyrA***	***AARS***	***WARS***	***gyrA***
Geo Mean [Ct]	18.33	25.28	18.85	15.66	17.76
Arithmetic Mean [Ct]	18.4	25.34	18.96	15.66	17.8
SD [± Ct]	1.36	1.52	1.87	0.45	1.02
CV [% Ct]	7.4	6	9.87	2.88	5.74
Min [x-fold]	-4.16	-4.83	-8.26	-1.65	-2.75
Max [x-fold]	5.80	4.69	5.13	1.85	3.75
SD [± x-fold]	2.57	2.87	3.66	1.37	2.03
Coeff. Of Corr. [*r*]	0.892	0.75	0.877	0.458	0.739
P value	0.003	0.032	0.004	0.254	0.036
Ranking	3	4	5	1	2

The ranking of the candidate reference genes using BestKeeper, GeNorm and NormFinder were not concordant. The geometric mean of the rankings from each of the algorithms was then applied in RefFinder with the sample result having the lowest value equalling the highest stability. Under NaCl conditions, *WARS* was most stable (1.73) with *gyrA* (4) and *AARS* (5) values high enough to pose questions about their overall stability. Under $MgSO_4$ conditions, *gyrA* demonstrated stable expression (1.68) followed closely by *WARS* (1.73).

For all algorithms explored in this study, *AARS* expression under NaCl conditions is the least reliable or stable of the candidate reference genes. *WARS* is the only gene that should be used as a reference

gene under NaCl conditions (based on GeNorm data), whereas both *gyrA* and *WARS* demonstrate suitability under $MgSO_4$ conditions. Gene expression results from *gyrA* and *WARS* under NaCl and $MgSO_4$ were combined and stability further assessed only with BestKeeper as GeNorm, NormFinder and RefFinder require 3 or more genes for analysis. Under the salt conditions tested *WARS* is the most stable (see Table 3 in body of article), with values generated for *gyrA* highlighting its unsuitability as a reference gene (cv % of 16.93).

Table S2. BestKeeper algorithm analysis of WARS and gyrA under both NaCl and $MgSO_4$ conditions.

Description	Gene stability under both NaCl and $MgSO_4$	
**Gene name**	***WARS***	***gyrA***
Geo Mean [Ct]	16.46	20.55
Arithmetic Mean [Ct]	16.50	20.9
SD [± Ct]	1	3.54
CV [% Ct]	6.03	16.93
Min [x-fold]	-2.87	-19.00
Max [x-fold]	6.09	124.36
SD [± x-fold]	1.99	11.62
Coeff. Of Corr. [*r]*	0.932	0.989
p-value	0.001	0.001

References

1. Haller, F.; Kulle, B.; Schwager, S.; Gunawan, B.; von Heydebreck, A.; Sültmann, H.; Füzesi, L . Equivalence test in quantitative reverse transcription polymerase chain reaction: Confirmation of reference genes suitable for normalization. *Anal. Biochem*. **2004**, *335*, 1–9.
2. Desroche, N.; Beltramo, C.; Guzzo, J. Determination of an internal control to apply reverse transcription quantitative PCR to study stress response in the lactic acid bacterium *Oenococcus oeni*. *J. Microbiol. Methods*. **2005**, *60*, 325–333.
3. Nieto, P.A.; Covarrubias, P.C.; Jedlicki, E.; Holmes, D.S.; Quatrini, R. Selection and evaluation of reference genes for improved interrogation of microbial transcriptomes: Case study with the extremophile *Acidithiobacillus ferrooxidans*. *BMC Mol. Biol*. **2009**, *10*, 63.
4. Guénin, S.; Mauriat, M.; Pelloux, J.; Van Wuytswinkel, O.; Bellini, C.; Gutierrez, L. Normalization of qRT-PCR data: The necessity of adopting a systematic, experimental conditions-specific, validation of references. *J. Exp. Bot*. **2009**, *60*, 487–493.
